# Personal

**Published:** 1912-01

**Authors:** 


### PERSONAL

DR. ROBERT C. KENNER of Louisville called on the editor last month—our first personal meeting after a friendship by correspondence lasting over twenty years. Dr. Kenner is the Editor of the *Therapeutic Record,* and a neurologist of note. Louisville has turned out over a third of all the physicians now in practice in the U. S. and still has more medical students than Chicago or New York. The relatively great increase in matriculants recently noted for the University of Buffalo, fortunately seems to be local; at any rate it is not observed in Louisville where the undergraduate medical students are about 800.

DR. JAMES S. PORTER of Buffalo, spent part of December in Toronto.

DR. J. F. VANDENBERG of Merritton, Ont., has been appointed surgeon to the Lincoln County Jail.

DR. ELIJAH JESSOP has been elected to a fifth term in the Ontario Parliament, for Lincoln Co.

MAJOR J. B. COAKLEY of Buffalo, has been appointed Civil Service Commissioner. He is a reconstructed Rebel of the finest type.

DR. CHARLES E. DE M. SAJOUS of Philadelphia, who formerly edited one of the best if not the best of the annual reviews of medical progress and whose work on the Ductless Glands is monumental, has been appointed Supervising Editor of the *New York Medical* JOURNAL. Mr. A. R. Elliott, President of the A. R. Elliott Publishing Co. says in a letter "We feel that you will extend to Dr. Sajous the good wishes, and to us the congratulations, that the occasion warrants." This is neatly and modestly worded and we concur most heartily.

MAJOR W. W. QUINTON, M. D., of Buffalo, Chairman of the Medical Committee on Social Evil, of the Buffalo Society of Sanitary Moral Prophylaxis, issues under date of November 15, 1911, a circular letter to the local profession to ascertain the prevalence of venereal diseases. If you have not replied, please do so, as a foundation of actual fact is needed for further work in the interests of a reform of inestimable importance.

Dr. James A. Gibson of Buffalo has an article on "Exploring the Thousand Islands" in the December issue of *Power Boating,* which took the hundred dollar first prize.

Dr. A. E. Bartoo, a University of Buffalo graduate of 1889, formerly of Arcadia, Neb., has lately been visiting relatives in Lackawanna and calling on his old friends in Buffalo, before sailing for Brazil where he expects to locate permanently .

Dr. Geo. W. Bachmann of Rochester, was recently the guest of Dr. A. E. Dean of Lackawanna.

Dr. Thomas J. Walsh of Buffalo, resigned the position of Examiner in Lunacy and his place was taken by Dr. B. Ross Nairn who, in turn, resigned the office of Medical School Examiner. As twenty Assistant Medical School Examiners were eligible to promotion, the matter was decided by lot and luck was chivalrous and gave the place to Dr. Christiana M. Greene, the only woman. The appointment is temporary, pending the establishment of an eligible list by examination.

Dr. Julius Y. Cohen of Buffalo, has been appointed to the vacancy due to the promotion of Dr. Greene, as above.

Dr. David Cohn of Buffalo, has been appointed Tuberculosis Inspector, vice Dr. Clarence Hyde, promoted to be Chief of the Tuberculosis Dispensary.

Dr. Francis E. Fronczak, Health Commissioner of Buffalo, is taking a trip to Havana, Cuba, Mexico and several South American Republics, partly for the sake of a rest well earned, partly to secure their co-operation in the International Congress of School Hygiene to be held in Buffalo in the summer of 1913.

Dr. Frederick Frisch, formerly of Buffalo, has removed to Atlantic City.

Dr. Frank H. Ransom of Buffalo, made a trip to Boston and New York in December.

Dr. Lucien Howe of Buffalo, has moved from the corner of Huron St. and Delaware Ave. to 520 Delaware Ave.

Dr. Clayton M. Brown of Buffalo, is contributing a series of articles on nursing in diseases of the eye, ear, nose and throat, to the Dietetic and Hygienic Gazette.

DR. GUY L. MCCUTCHEON of Buffalo has moved to 520 Potomac Ave., corner of Claremont Place.

---

DR. GROVER W. WENDE of Buffalo, presented a paper before the Dermatological section of the Rochester Academy of Medicine on December 13.

---

DR. J. L. ROSEBOOM of Rochester, spoke on "Esperanto as the universal language of Science" at the Rochester Academy of Science, December 11.

---

DR. JAMES F. SHERMAN (Buffalo 1885) of Rochester was operated on and has made a good recovery.

---

DR. M. A. KING of Rochester, has also made a complete recovery and has just removed to 21 East Avenue.

---

THE appointment, by Mayor Baker, of Dr. F. D. Crim as Health Officer, and Dr. F. R. Ford as deputy health officer for Utica, gives very general satisfaction.

Dr. Crim has been in general practice for about thirty years, is a physician of excellent attainments and repute, and in every way well qualified for the head of our health department.

Dr. F. R. Ford is especially well fitted for the position of deputy, being an eminent bacteriologist, and having served one term in this position very acceptably with Dr. Peck as health officer.

---

THE home of Dr. A. H. Baker (Buffalo '85) of Elmira, was partially destroyed by fire the night of December 18th. The damage was about $2,000, covered by insurance. Dr. Baker's office was not damaged.

---

DR. PARKER SYMS invites the profession to his surgical clinics at Lebanon Hospital, New York, Wednesdays, 3 P. M. till May 1.

